# Inner Segment Ellipsoid Band and Cone Outer Segment Tips Changes Preceding Macular Hole Development in a Young Patient

**DOI:** 10.1155/2014/132565

**Published:** 2014-12-04

**Authors:** Mariana Harasawa, Hugo Quiroz-Mercado, Guillermo Salcedo-Villanueva, Gerardo Garcia-Aguirre, Shulamit Schwartz

**Affiliations:** ^1^Department of Ophthalmology, University of Colorado School of Medicine, Denver Health Medical Center, Denver, CO 80204, USA; ^2^Rocky Mountains Lions Eye Institute, University of Colorado School of Medicine, Aurora, CO, USA; ^3^Association to Prevent Blindness in Mexico (APEC), Hospital Dr. Luis Sanchez Bulnes, 04030 Coyoacan, DF, Mexico

## Abstract

*Purpose*. Pathophysiology of macular hole (MH) is not yet well defined but the advances of spectral domain optical coherence tomography (SD-OCT) give us access to further detailed imaging. We report a case with macular inner segment ellipsoid (ISe) band loss and cone outer segment tips (COST) line changes seen in SD-OCT preceding MH appearance in a young patient. *Methods*. 21-year-old woman presented with a partial central scotoma, metamorphopsia, and a 20/25 vision in her right eye. Past medical history was positive for laser assisted in situ keratomileusis (LASIK) surgery 7 months ago with no complications. Macular SD-OCT showed ISe band loss and COST line elevation. She was followed a month later with visual acuity deteriorating to 20/200 and a full thickness MH. *Results*. The patient underwent a pars plana vitrectomy with internal limiting membrane peeling. Her visual acuity 2 months later was 20/20. *Conclusion*. SD-OCT can identify preliminary changes, yet to be described, preceding MH formation. Our patient demonstrated ISe band loss and COST abnormalities on SD-OCT a month prior to MH development. SD-OCT should be considered in young patients with subtle visual symptoms and mild changes in visual acuity that are not readily explained by ophthalmological exam.

## 1. Introduction

Macular hole (MH) is an idiopathic condition that in most cases is related to posterior vitreous detachment (PVD) and is mainly seen in the elderly. The minority of cases are observed in younger patients with a history of blunt ocular trauma as well as in myopic patients after laser assisted in situ keratomileusis (LASIK) surgery [1, 2].

Spectral domain optical coherence tomography (SD-OCT) provides further detailed imaging that allows the detection of early anatomical changes, enabling us to alter and/or refine current theories.

Different pathophysiological mechanisms might be involved in MH formation depending on vitreous syneresis and posterior hyaloid status. Several theories have emerged involving tangential traction [3] and anterior-posterior traction [4, 5]. The exact pathogenesis remains unclear but it is broadly believed to have a strong relationship with the vitreous traction exerted on the fovea. As a consequence, several anatomical changes have been described as the primary findings in the macular hole development such as intraretinal split, foveal pseudocyst, and foveolar detachment [5–7].

We report a case of a young patient who at first presented with subtle visual symptoms on the right eye. Her SD-OCT showed changes in the inner segment (ISe) band and cone outer segment tips (COST) line preceding MH formation in the absence of any vitreomacular interface abnormality in the ophthalmological examination.

## 2. Material and Methods

**Figure 1:**
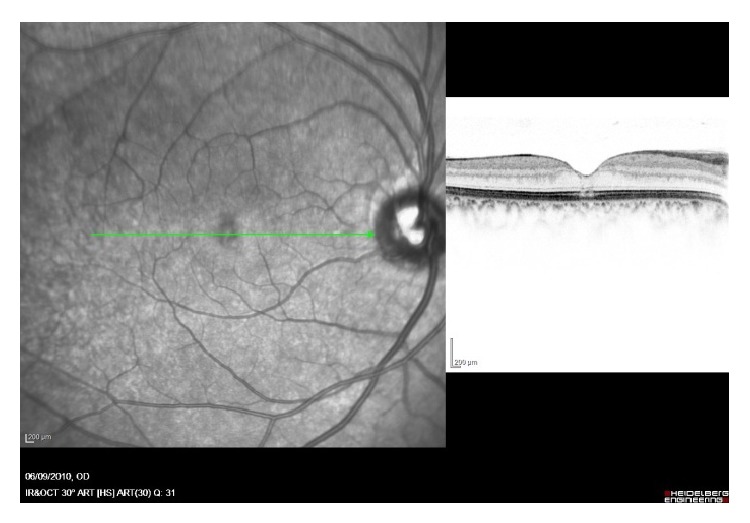
Spectral domain optical coherence tomography (Spectralis; Heidelberg Engineering, Heidelberg/Germany) of the right eye. Subfoveal inner segment band loss and cone outer segment tips elevation.

**Figure 2:**
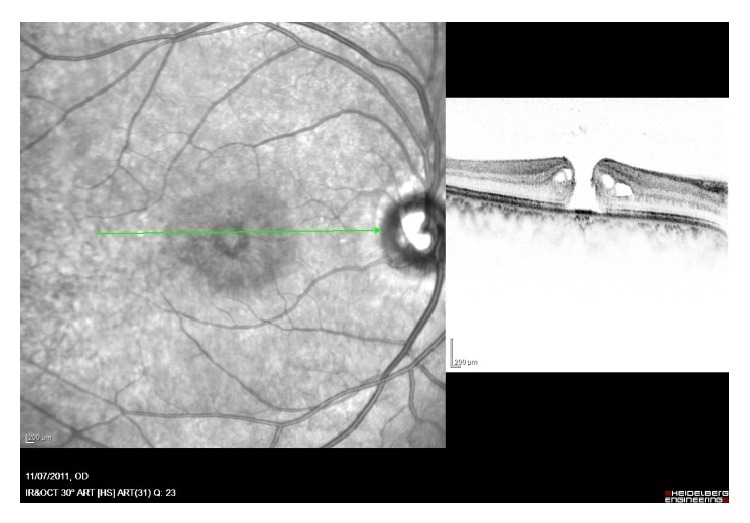
Spectral domain optical coherence tomography (Spectralis; Heidelberg Engineering, Heidelberg/Germany) of the right eye. Full thickness macular hole and no evidence of posterior vitreous detachment (stage 3).

A 21-year-old female patient presented to the retina clinic with a new onset of partial central scotoma and metamorphopsia in her right eye (RE). Best corrected visual acuity (BCVA) of the RE was 20/25. Left eye (LE) was asymptomatic with BCVA of 20/20. She had no history of trauma and reported having a successful LASIK surgery in both eyes seven months earlier with BCVA of 20/20 in both eyes after surgery. She had a positive ocular family history, with her father being operated for a unilateral idiopathic MH. Dilated fundus examination did not reveal any retinal findings in both eyes. Therefore, a SD-OCT (Spectralis; Heidelberg Engineering, Heidelberg/Germany) was performed and showed an ISe band loss and COST line changes at the subfoveal area with no other findings in the RE (Figure 1). She was followed a month later complaining of decreased vision on her RE and a larger central scotoma. BCVA of the RE was 20/200 and dilated eye exam showed a full thickness macular hole (FTMH). On SD-OCT, there was a stage 3 macular hole (Figure 2). The LE had a BCVA of 20/20, no apparent abnormalities on examination, and intact ISe band and COST line on SD-OCT.

## 3. Results

**Figure 3:**
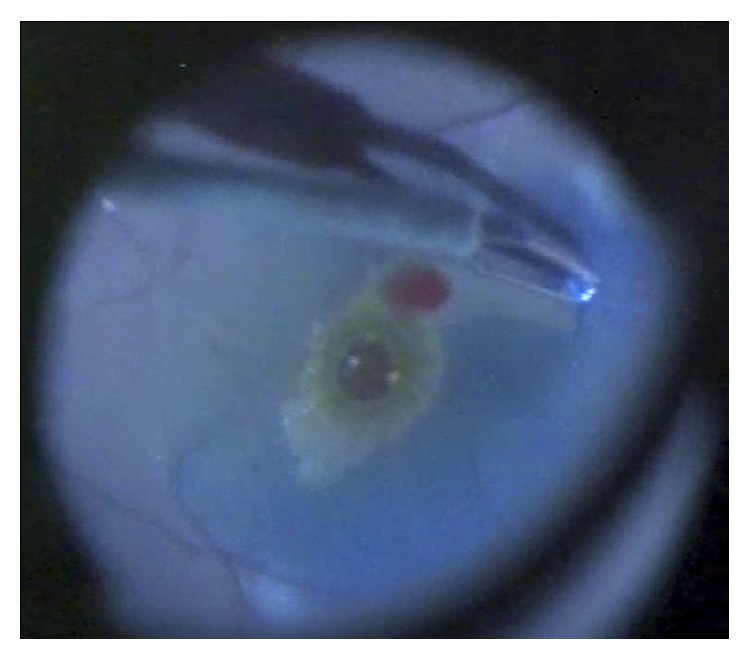
Internal limiting membrane peeling during right eye pars plana vitrectomy after brilliant blue staining. Note: edge of internal limiting membrane peeling (black arrow) and full thickness macular hole (white arrow).

A 23-gauge pars plana vitrectomy was performed in the RE. The posterior hyaloid was found to be very adherent and the surgeon faced difficulties trying to induce a PVD using high aspiration with the vitrector probe. A brilliant blue stain was injected to facilitate the visualization of the internal limiting membrane (ILM) on the margins of the macula (Figure 3). The ILM was identified and eventually peeled en bloc with the posterior hyaloid from an edge close to the foveal center. Gas tamponade with SF6 12% was used at the end of the operation with the patient facing down. The patient's BCVA two months after the operation was 20/20 with no residual symptoms.

## 4. Discussion

Gass studied the pathophysiology of MH and suggested that the majority of macular holes begin as a central dehiscence with little loss of retinal tissue following tangential traction [8]. Biomicroscopy usually detects a foveal pseudocyst, radial striae, a yellow spot or ring, or a combination of these findings in stage 1 impending hole. On OCT, the cystoid space occupies the inner part of the foveal tissue and can extend posteriorly to disrupt the outer retinal layers [7].

Studies by SD-OCT showed minor changes in the outer foveolar structure, especially in the COST line, in either eyes with a foveolar yellow spot or eyes with vitreomacular traction [9, 10]. We showed an interruption of the retinal tissue at the level of the ISe and COST line on SD-OCT a month before FTMH appearance, but our case did not show any abnormality in ophthalmoscopy. These mild changes were detected due to the patient's complaint of a discrete central scotoma with minimal visual impairment that could be imperceptive to other patients. Moreover, our patient did not have a PVD on examination with a very adherent posterior hyaloid during vitrectomy. Uemura et al. found that a focal vitreomacular attachment with anteroposterior traction on the foveal surface plays an important role in the elevation of the COST line. They observed that the elevation of the COST layer spontaneously resolved after vitreomacular separation indicating a strong relationship between elevation of COST line and vitreomacular traction [11].

We speculate that the strong vitreomacular adherence observed during vitrectomy might have induced vitreoretinal interface changes that caused changes in the outer retina with no apparent signs of traction on ophthalmoscopy. In patients with a strong vitreomacular adhesion, ISe band loss and COST line changes on SD-OCT may be a harbinger for MH formation; these patients should be followed closely. We are unaware of any publications on OCT changes preceding MH formation in young patient as we described with normal fundus examination.

There is a higher incidence of MH in patients with a positive familial history of MH, suggesting the involvement of genetic factors [12]. Our patient had a father with a diagnosis of idiopathic MH that might also imply her MH formation.

Although there are reports of MH cases after LASIK surgery [2], we believe that this case was not related to the prior LASIK surgery because of the 7-month interval between surgery and MH formation.

In conclusion, SD-OCT can identify preliminary changes at the level of the foveal ISe band and COST line preceding MH formation. Therefore, it should be considered in young patients with subtle visual symptoms and mild changes in visual acuity that are not readily explained by ophthalmological examination.
